# The immune response as a therapeutic target in non-alcoholic fatty liver disease

**DOI:** 10.3389/fimmu.2022.954869

**Published:** 2022-10-10

**Authors:** Nicolás Ortiz-López, Catalina Fuenzalida, María Soledad Dufeu, Araceli Pinto-León, Alejandro Escobar, Jaime Poniachik, Juan Pablo Roblero, Lucía Valenzuela-Pérez, Caroll J. Beltrán

**Affiliations:** ^1^ Laboratory of Immunogastroenterology, Unit of Gastroenterology, Department of Medicine, Hospital Clínico Universidad de Chile, Santiago, Chile; ^2^ School of Medicine, Faculty of Medicine, Universidad de Chile, Santiago, Chile; ^3^ Faculty of Dentistry, Universidad de Chile, Santiago, Chile; ^4^ Unit of Gastroenterology, Department of Medicine, Hospital Clínico Universidad de Chile, Santiago, Chile

**Keywords:** non-alcoholic fatty liver disease (NAFLD), microbiota-gut-liver axis, low-grade inflammation, liver lymphocyte homing, steatohepatitis (NASH), microbiota, liver diseases, liver fibrosis

## Abstract

Non-alcoholic fatty liver disease (NAFLD) is a complex and heterogeneous disorder considered a liver-damaging manifestation of metabolic syndrome. Its prevalence has increased in the last decades due to modern-day lifestyle factors associated with overweight and obesity, making it a relevant public health problem worldwide. The clinical progression of NAFLD is associated with advanced forms of liver injury such as fibrosis, cirrhosis, and hepatocellular carcinoma (HCC). As such, diverse pharmacological strategies have been implemented over the last few years, principally focused on metabolic pathways involved in NAFLD progression. However, a variable response rate has been observed in NAFLD patients, which is explained by the interindividual heterogeneity of susceptibility to liver damage. In this scenario, it is necessary to search for different therapeutic approaches. It is worth noting that chronic low-grade inflammation constitutes a central mechanism in the pathogenesis and progression of NAFLD, associated with abnormal composition of the intestinal microbiota, increased lymphocyte activation in the intestine and immune effector mechanisms in liver. This review aims to discuss the current knowledge about the role of the immune response in NAFLD development. We have focused mainly on the impact of altered gut-liver-microbiota axis communication on immune cell activation in the intestinal mucosa and the role of subsequent lymphocyte homing to the liver in NAFLD development. We further discuss novel clinical trials that addressed the control of the liver and intestinal immune response to complement current NAFLD therapies.

## Introduction

Non-alcoholic fatty liver disease (NAFLD) is a liver disorder characterized by fat accumulation in at least 5% of the liver cells in individuals without significant alcohol consumption (1) and no secondary causes of chronic liver disease such as hepatitis, medications, toxicants in the environment, parenteral nutrition, Wilson’s disease, and chronic liver disease (hemochromatosis, autoimmune liver disease, chronic viral hepatitis, fatty liver of pregnancy, and tyrosinemia) (2). NAFLD has become a term encompassing a clinicopathological spectrum ranging from simple steatosis to non-alcoholic steatohepatitis (NASH), the more severe form of NAFLD that can lead to advanced fibrosis and cirrhosis. The global prevalence of NAFLD has increased over the last decades; a recent meta-analysis that included 363 studies from 40 countries or regions worldwide reported a pooled estimated prevalence of 29.38% (3). A population-based observational study including 21 regions and 195 countries reported a rise in prevalence from 8.2% in 1990 to 10.9% in 2017, demonstrating a global public health problem (4). NAFLD is commonly related to metabolic syndrome, which in turn is characterized by an increase in the risk of cardiovascular disease (CVD) (5). Interestingly, CVD is the leading cause of NAFLD-related deaths after cirrhosis (6). This evidence emphasizes the need for broad clinical management of the disease to reduce the associated cardiovascular risk.

Despite increasing advances in the understanding of the pathophysiology of NAFLD, the exact mechanisms involved in the progression towards liver damage remain unknown. The “two-hit” hypothesis has been postulated to explain the pathogenesis of NAFLD. Here, the first event or “hit” denotes an increase in lipolysis and the consequent accumulation of triglycerides in the liver (7). The second “hit” is subsequently generated by an imbalance between reactive nitrogen and reactive oxygen species, leading to increased inflammatory injury that is accompanied by the release of various cytokines that contribute to hepatocellular injury and fibrosis (8). In addition to this hypothesis, a decade ago, an alternative model of multiple parallel hits emerged that includes various factors, such as the interaction between the gut microbiota and immune system, that promote the progression from simple steatosis to NASH; these factors have not been wholly described thus far (9).

Elevated immune cell activation has been widely described in the pathophysiology of NAFLD (10). However, the main studies have been centered on evaluating the role of these inflammatory processes in the liver; the activation of the immune response in the gut and its impact on liver function are poorly described. The intestine comprises one of the most oversized compartments of the immune system that is continually exposed to antigens from the diet and the microbiota (11). The balance in the adequate activation of the immune response, either to pathogenic luminal antigens or to commensal tolerogenic stimuli, determines the type of activation of a chronic inflammatory response that could promote liver damage.

This review aims to provide an integrated overview of the current state of knowledge on the role of the immune response in NAFLD. We mainly focused on the innate and adaptive mucosal activation of the intestinal immune response in this disease as a part of the altered communication of the microbiota-gut-liver axis. In addition, based on this knowledge, we expose possible therapeutic targets directed at controlling hepatic and intestinal inflammation and adaptive immune responses.

## The microbiota-gut-liver axis in the pathogenesis of NAFLD

The pathogenesis of NAFLD is complex and multifactorial, including genetic predisposition, obesity, insulin resistance, increased immune response, altered gut microbiota, and environmental factors such as diet. These factors configure metabolic syndrome, which is characterized by increased serum-free fatty acid, triglycerides, LDL, and total cholesterol and decreased HDL levels, as well as adipocyte dysfunction. The excess of free fatty acid in the liver leads to steatosis and lipotoxicity that induce mitochondrial dysfunction, oxidative stress, and endoplasmic reticulum stress (12). Accumulated evidence suggests that this oxidative stress process and consequent liver damage through activation of the liver immune response plays an important role in the pathogenesis of NAFLD (13). As result, the activation of these processes leads to a systemic low-grade inflammation with increased levels of cytokines such as tumor necrosis factor (TNF)-α and interleukin (IL)-6, proposed as inflammatory markers of NAFLD (14).

**Figure 1:**
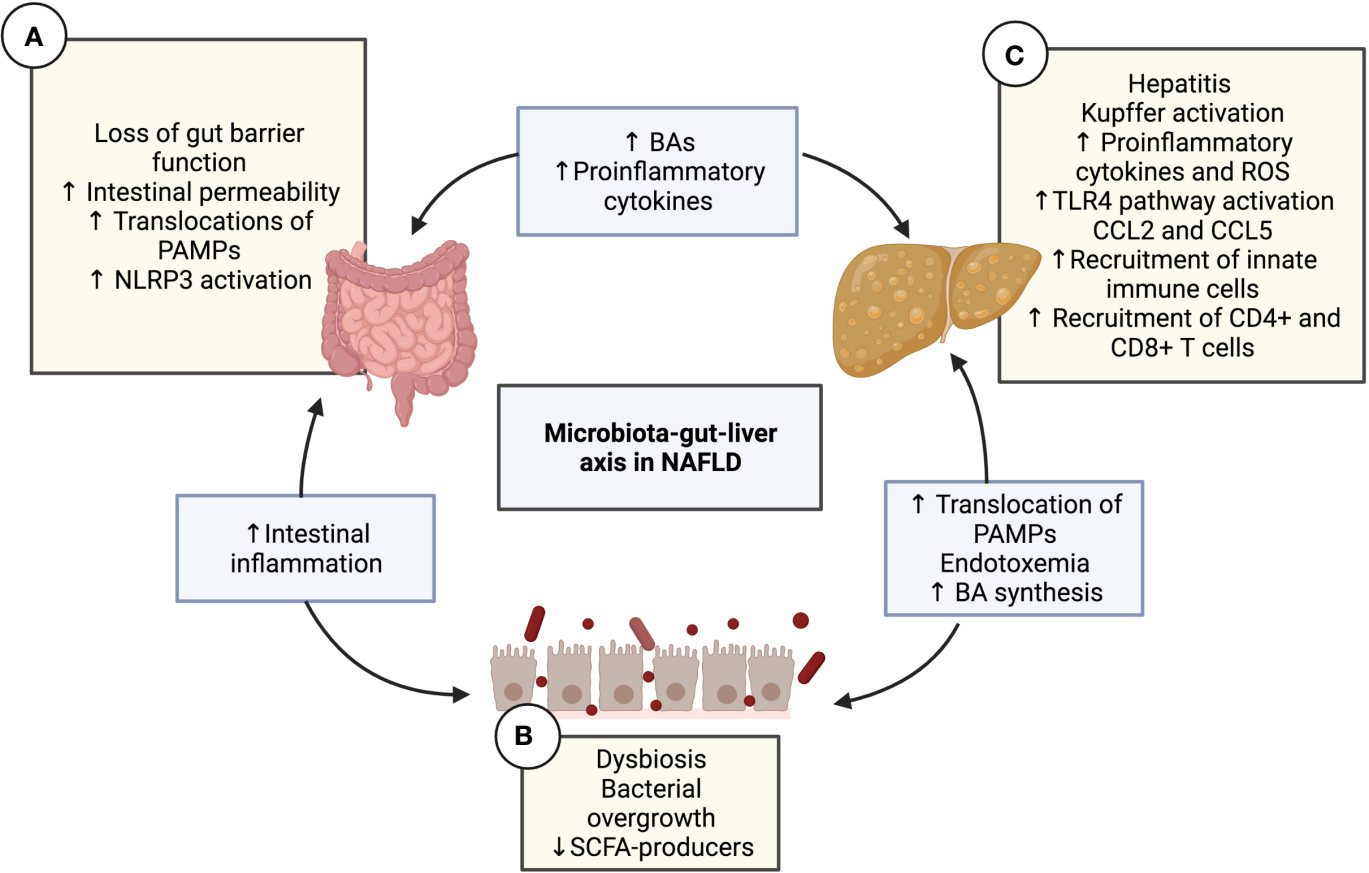
The microbiota-gut-liver axis in NAFLD. Interaction diagram of the different mechanisms of the microbiota-gut-liver axis participating in the pathogenesis of NAFLD. **(A)** Intestinal gut barrier disruption and increased permeability have been demonstrated in patients with NAFLD along with the decreased expression of junctional adhesion molecule A, zonula occludens-1, and occludin. This alteration causes the transfer of pro-inflammatory products and PAMPs (such as LPS or PGN) to the liver circulation, configuring intestinal inflammation and endotoxemia. The translation of PAMPs causes TLR signaling in the mucosa, which leads to the activation of NLRP3. **(B)** Diet nutrient composition can affect the quantitative and qualitative composition of the gut microbiota, leading to intestinal dysbiosis and bacterial overgrowth, which impacts the immune response, favoring NAFLD progression. Dysbiosis contributes to the disruption of the intestinal barrier, increasing mucosal permeability, which produces more dysbiosis, thereby creating a vicious cycle. Another consequence of dysbiosis is the alteration in the homeostasis of microbe-derived metabolites, such as a decrease in SCFAs and an increase in BAs. **(C)** The liver is a vital organ in fat metabolization and undergoes many changes in patients with metabolic syndrome, including the over-accumulation of free fatty acid, activation of KCs due to and the TLR4 pathway, lipotoxicity, increased reactive oxygen species and cytokines, and finally, steatosis. Hepatic CCL5 expression levels have been shown to increase in NAFLD patients. The release of the chemokines CCL2 and CCL5 is crucial in the recruitment of lymphocytes to the liver. The migration of mesenteric lymph node cells into the liver is mediated by CCL5, which induces hepatic CD4+ T and CD8+ T cell activation, subsequently leading to liver injury and the progression of NAFLD. BA, Bile acid; CD, Cluster of differentiation; CCL, C-C motif chemokine ligand; KC, Kupffer cell; LPS, Lipopolysaccharide; NAFLD, Non-alcoholic fatty liver disease; NLRP3, NLR family pyrin domain containing 3; PAMP, pathogen-associated molecular pattern; PGN, Peptidoglycan; ROS, Reactive oxygen species; SCFA, Short-chain fatty acid; TLR, Toll-like receptor.

In 1982, a positive correlation between intestinal bacterial overgrowth and hepatic steatosis was observed in patients, being the first evidence of the role of the intestinal microbiota in hepatic steatosis (15). Since then, the influence of the intestinal microbiota on the development of liver disease has been highlighted. Many research works have focused on studying NAFLD pathogenesis regarding the interaction between the gut microbiota and liver function (**Figure 1**). These studies even investigated the intermediary role of the gut immune system in the interplay between the microbiota and liver function. In this regard, it has been observed that microbiota composition is significantly influenced by genetic and environmental factors, such as diet, which induce metabolic changes in the activity of the gut microorganisms. The metabolites released by the microbiota promote inflammatory processes that contribute to the pathogenesis of the disease (16). Therefore, the interaction among the components of the microbiota-gut-liver axis defines the behavior of diverse dependent mechanisms, such as intestinal barrier function, systemic immune responses, and hepatic inflammation, all of which are seriously altered in NAFLD.

A loss of intestinal homeostasis induces changes in the diversity and composition of the gut microbiota that impact the mucosal immune response and promote NAFLD progression. Likewise, bacterial overgrowth in the small and large intestine has been observed in patients with NAFLD (17, 18), which has been associated with impairment of the intestinal barrier functions and an activated intestinal immune response in an NAFLD/NASH mouse model (19). In this regard, it is important to consider that the intestinal barrier controls the transport of substances from the gut to the enterohepatic circulation by preventing the translocation of pathogens and molecules, such as pathogen- and damage-associated molecular patterns (PAMPs and DAMPs). Its function involves diverse components, among them the mucus lining and an epithelial monolayer of specialized cells that are bound by junctional complexes. These complexes include tight junctions (TJs), which play a sealing role in the intercellular space to control paracellular passage (20). Increased intestinal permeability and elevated levels of inflammation are positively correlated with the onset and progression of NAFLD (21). Altered gut barrier function in this disorder is related to decreased expression of the TJ proteins zonula occludens-1 (ZO-1) and occludin (22). Under normal conditions, the liver exerts an immune vigilance role by supporting the clearance of bacterial products from the portal circuit. However, when intestinal barrier function is altered, LPS and other bacteria-derived compounds rise in the circulation, increasing the activation of toll-like receptors (TLRs) and other pathogen recognition receptors (PRRs) in the liver, thereby triggering inflammatory responses in this organ (23). Several studies have shown higher LPS content in the serum as well as hepatocytes in NAFLD patients (24, 25). In the liver, through its interaction with TLR4 expressed on resident hepatic macrophages known as Kupffer cells (KCs), LPS triggers a signaling pathway toward activating nuclear factor kappa B (NF-κB), which promotes the expression of pro-inflammatory cytokine genes such as IL-6. A higher number of TLR4-positive macrophages were observed in NASH patients in comparison to patients with simple steatosis and controls, where TLR4 expression was positively correlated with serum LPS levels (24). These data support that the restoration of intestinal permeability *via* microbiota modulation can be an attractive therapeutic target for NAFLD (26).

Intestinal dysbiosis induces alterations in the produced microbe-derived metabolites including short-chain fatty acids (SCFAs) and secondary bile acids (BAs). The role of each in NAFLD is described below.

### The role of SCFAs in NAFLD

SCFAs are produced by anaerobic bacterial fermentation that influences the intestinal epithelial barrier function and immune response. It has been shown that SCFAs modulate the differentiation of several immune cells, such as macrophages, dendritic cells (DCs), and T regulatory cells (Tregs), and contribute to the control of some immune cell functions, like the phagocytic activity of macrophages (27). In this regard, murine colonic macrophages treated with oral butyrate, one of the most common SCFAs in the human intestine, enhanced their antimicrobial activity without an increased inflammatory cytokine response, suggesting that increased intestinal butyrate might represent a strategy to bolster host defenses without damaging tissue inflammation (28). Additionally, it has been demonstrated that butyrate, as well as the other common SCFAs acetate and propionate, can directly promote T cell differentiation into CD4^+^ T cells producing IL-17, interferon-γ, or IL-10, depending on the cytokine milieu (29). This evidence suggests that the intestinal microbiota, through the production of SCFAs, exerts a regulatory role on the immune response. *In vitro* studies in several cell types, including monocytes, macrophages, and KCs, have demonstrated that SCFAs suppress the LPS and cytokine-stimulated production of proinflammatory mediators, such as TNF-α, IL-6, and NO (30). Currently, only evidence based on animal models is available; a high-fat diet (HFD) mouse model study showed that the intragastric administration of sodium butyrate ameliorated HFD-induced hepatic steatosis, inflammation, and gut microbiota imbalance in the mice (31). In summary, these findings suggest that SCFAs regulate the development of inflammatory responses in NAFLD, have important anti-inflammatory activity, and may have a beneficial effect. Further research is necessary to determine the specific mechanism by which SCFAs affect the occurrence and development of NAFLD.

### The role of BAs in NAFLD

The microbiota-gut-liver axis is essential to regulating systemic metabolism (32). BAs are steroid-derivative components of bile that participate in communication along this axis. They have a significant role in many physiological processes, such as the digestion and solubilization of lipids, the regulation of hepatic glucose levels, and inflammation (33). Under diverse pathological conditions, such as NAFLD, the size of the BA pool and its composition are altered. In patients with NAFLD, the serum’s total, primary, and conjugated BAs are significantly increased, with slight changes in unconjugated BAs. However, secondary BAs were observed to considerably increase in some studies and decrease in others (34). Thus, there is no clear consensus among the studies of hepatic BAs in patients with NAFLD. Despite these contradictory findings, limited clinical studies concluded that hepatic BA homeostasis is dysregulated in this pathology (34), which can be associated with alterations in the regulation of BA homeostasis by the dysbiotic intestinal microbiota. Increased intestinal permeability is associated with alterations in BA composition as well as metabolic endotoxemia and inflammation, which are common findings in patients with NAFLD (35). A study by Gupta et al. in a murine model of NAFLD demostrated that the use of sevelamer hydrochloride to sequester intestinal BAs decreased mucosal inflammation and improved intestinal barrier function. This was correlated with reduced liver injury and reduced hepatosteatosis, demonstrating the therapeutic potential of targeting BAs in NAFLD (36). This evidence suggests that the modulation of BAs and the microbiota can be a good therapeutic target in NAFLD.

In addition, impaired BA signaling has been shown to be an essential mechanism for NAFLD development (37). Through interaction with one of the BA receptors, the farsenoid X receptor (FXR), BA can increase insulin sensitivity and decrease hepatic gluconeogenesis and circulating triglycerides (38). In this regard, Yang et al. observed lower levels of hepatic FXR and elevated triglyceride levels in patients with NAFLD compared to normal controls (39).

Despite evidence that BA can directly modulate both innate and adaptive immune cell responses (40, 41), its role in NAFLD immunity is not well-explored. Diverse BA receptor agonists have attracted the attention as drug candidates for intestinal inflammation (42, 43); however, there are no clinical trials evaluating the targeting of this mechanism in NAFLD.

## Gut mucosal immunity in NAFLD

The mucosa of the gastrointestinal tract (GIT) is the most extensive area of the immune system in the human body. Microbial colonization of the GIT in early life is crucial to the proper education and maturation of immune cells since it will determine an effective response against pathogens and tolerance to commensal antigens. The gut mucosal immune response occurs in two different compartments, namely the inductive and effector sites. The first site, in which the adaptive immune response occurs with the priming and differentiation of lymphocytes, includes the mesenteric lymph nodes (MLNs) and the gut-associated lymphoid tissues (GALT) consisting of Peyer’s patches (44) and isolated lymphoid follicles (ILFs), distributed along the small and large intestines (45). The second site comprises the epithelium and intestinal lamina propria, in which immune cells are located and activated to promote intestinal barrier functions (44). In a coordinated manner, both innate and adaptive responses are exerted at the two mucosal sites to respond against pathogenic insult and commensal stimuli.

### Innate immune response

**Figure 2:**
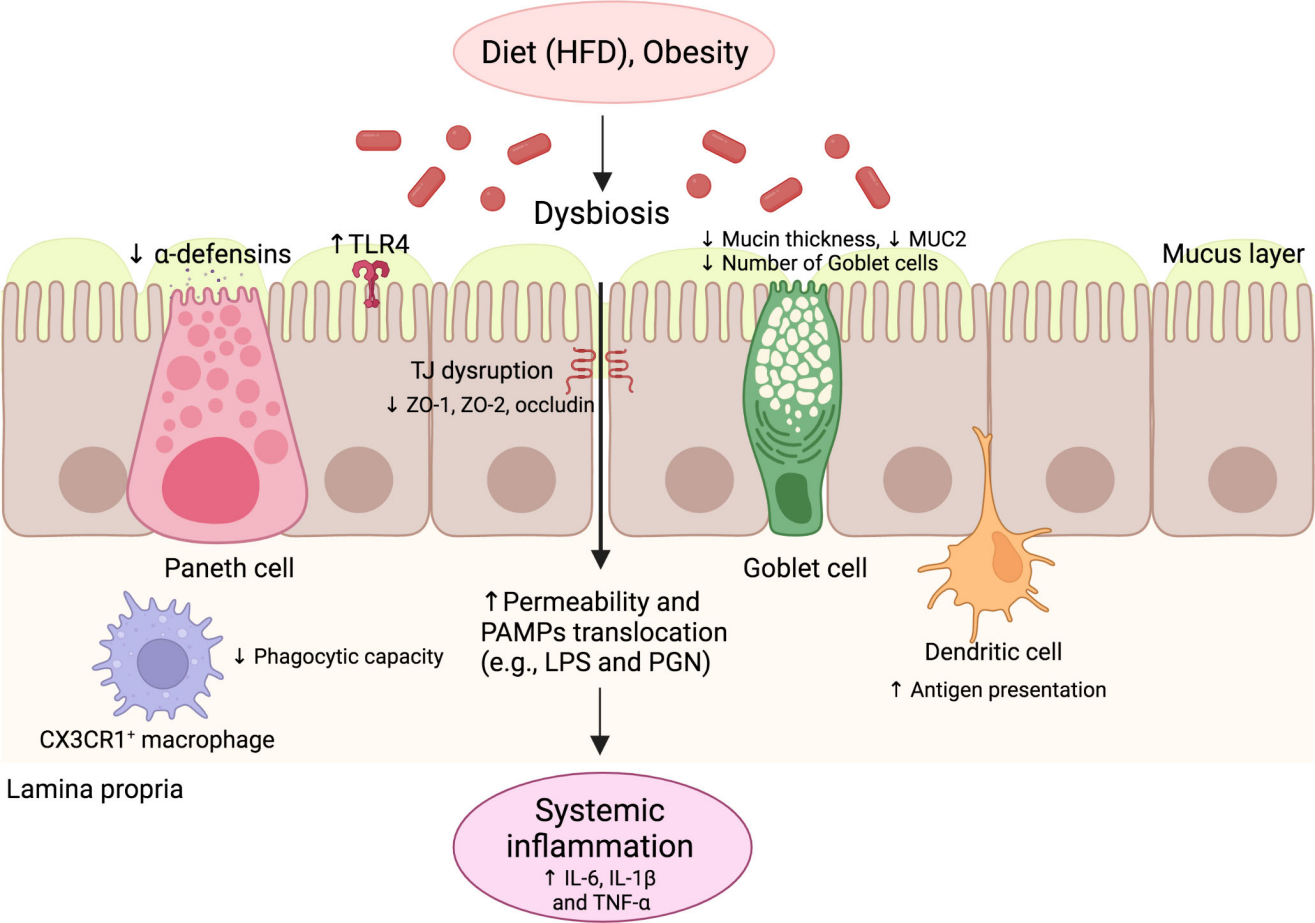
The innate immune response of the gut in NAFLD. Environmental factors such as diet and obesity promote dysbiosis in the gut microbiota, leading to increased levels of PAMPs and impaired intestinal barrier function. The intestinal barrier dysfunction is characterized by the impaired function of several cells, such as goblet cells and Paneth cells, with decreased production of mucin and antimicrobial peptides, respectively. TJ structure and composition disruption also occurs, characterized by reduced ZO-1, ZO-2, and occludin expression. Additionally, alterations of antigen-presenting cell function occur, including decreased phagocytic capacity and increased antigen presentation. These alterations promote increased intestinal permeability and the translocation of PAMPs, leading to increased serum levels of pro-inflammatory cytokines and systemic inflammation. HFD, High-fat diet; IL, Interleukin; LPS, Lipopolysaccharide; NAFLD, Nonalcoholic fatty liver disease; PAMP, pathogen-associated molecular pattern; PGN, Peptidoglycan; TLR, Toll-like receptor; TNF, Tumor necrosis factor.

Oral substances absorbed in the intestine reach the liver *via* the portal circulation, continuously exposing this organ to potential antigens. Liver damage can be initiated and enhanced by a local intestinal immune response whose activation promotes inflammation and the migration of immune cells to the liver. The leaky gut processes contribute to intestinal inflammation (**Figure 2**). The loss of epithelial barrier integrity increases the translocation of microbial components to the lamina propria and liver, activating receptors that initiate signaling conducted by the gene expression of diverse elements of innate immunity. Systemic inflammation contributes to the pathogenesis of NAFLD, characterized by a high number of neutrophils and macrophages in the liver (46). This response leads to liver cell death that supports the disease’s progression (47). Moreover, diverse resident liver cells, like parenchymal hepatocytes and lymphoid and non-lymphoid cells play an essential role in the homeostasis of the liver immune response, apart from being involved in modulating NAFLD progression (48).

#### Intestinal barrier

The intestinal epithelium is a single layer of luminal lining cells in the gut, considered the most significant communication barrier between the internal and external environments (49). The intercellular surface in the epithelium includes diverse junctional complexes, among them desmosomes, adherens junctions (AJs), and TJs. While AJs and desmosomes are essential for the mechanical linkage of adjacent cells (49), TJs play a sealing role that controls the paracellular transport of luminal agents towards the lamina propia (49). It has been observed that the composition of TJ proteins in the small intestine is altered in NAFLD, with decreased expression of ZO-1 (21). As already mentioned, the intestinal microbiota influences intestinal epithelial integrity. In the HFD mouse model, oral treatment with *Faecalibacterium prausnitzii*, considered a bacterial indicator of a healthy gut, significantly increased the expression of the intestinal TJ protein-encoding Tjp1 gene that encodes the ZO-1 protein (50). Similarly, Briskey et al. showed significant liver steatosis and reduced expression of TJ proteins such as ZO-1 and ZO-2 in an HFD mouse model (51). Interestingly, in this study, probiotic supplementation mitigated the severity of steatosis by partially preventing TJ expression, suggesting that the modulation of TJs is a good strategy to reverse the progression of steatosis in NAFLD.

Other components of the intestinal barrier include the goblet cells and Paneth cells, which can secrete mucins that are part of the intestinal mucus (52) and antimicrobial peptides (53) that control bacterial load at the lumen (54, 55), respectively. These cells are in close interaction with a sizeable population of immune cells immersed in the epithelial layer, such as intraepithelial lymphocytes, which contribute to the first line of defense in the gastrointestinal mucosa (56). An alteration in the number and function of these cell components has been described in animal models of obesity and NAFLD patients. HFD-fed mice showed a decreased number of goblet cells in the ileal crypts and intestinal MUC2 expression (57, 58). These alterations were reversed through the administration of nuciferine, a bioactive component derived from the lotus leaf that could have a protective role in the epithelial layer (57). Additionally, resistin-like molecule β (RELMβ), expressed in the secretory granules of intestinal goblet cells, has been described to regulate gut microbiota composition, contributing to the maintenance of immune response the gut homeostasis. Increased intestinal expression and serum concentrations of RELMβ, which promote insulin resistance, have been observed in HFD-fed mice (59). Furthermore, RELMβ knockout mice were resistant to a methionine- and choline-deficient (MCD) diet, suggesting the contribution of increases in RELMβ to NASH development and raising the possibility that RELMβ is a novel therapeutic target for this pathology (60).

Paneth cells produce defensins (α and β), cathelicidins (LL-37/CRAMP), and C-type lectins (RegIII α/γ/β), which constitute the AMP gut repertoire. These AMPs control the interaction of the gut microbiota with the intestinal mucosa. Mice lacking RegIIIγ have been shown to display an altered mucus distribution that increases the proximity of microbiota to the intestinal epithelium, inducing inflammation in the ileal mucosa. As AMP promotes barrier integrity, a low number of Paneth cells impairs a proper bacterial defense and favors NAFLD progression (61). Moreover, the depletion of Paneth cells’ granules by intravenous dithizone, a zinc chelating agent, has been observed to ameliorate the severity of NAFLD in HFD-fed mice; this effect was associated with changes in gut microbiota composition (62). Furthermore, insufficiency of vitamin D is considered one of the risk factors for metabolic syndrome and NAFLD, which is associated with the decreased production of Paneth cell defensins. In this regard, HFD-fed mice with vitamin D deficiency (HFD+VDD) showed a decrease in the ileum-specific α-defensins of Paneth cells, associated with increased intestinal permeability and gut dysbiosis (58). In the same study, the oral administration of α-defensin-5 in the HFD+VDD model restored the eubiotic state, decreasing *Helicobacter hepaticus*, a bacteria that causes hepatitis and liver tumors in mouse models, and increasing *Akkermansia muciniphila*, a symbiotic bacteria, that restored metabolic disturbances (i.e., glucose levels, body mass, and liver fat content) (58). This evidence suggests a protective role for Paneth cell defensins in NAFLD development, mediated by the modulation of gut microbiota composition.

#### Pattern recognition receptors

A delicate interplay between the gut microbiota, epithelium, and immune cells in the mucosa allows for the maintenance of selective permeability in the intestine (Kolodziejczyk, 2019). PRRs, such as TLRs and NLRs, are responsible for recognizing molecular patterns inducing inflammatory responses that are crucial in the pathogenesis of NAFLD. The intestinal epithelial cells express several TLRs, including TLR1, TLR2, TLR4, TLR5, and TLR9 (63). Regarding the role of intestinal PRRs in NAFLD, upregulated signal activity of TLR4 has been observed in HFD-fed mice, which was associated with the elevated transcription of inflammatory cytokines such as IL-6 and IL-1β (64). TLR4 is expressed in immune cells, mainly of myeloid origin, including monocytes, macrophages, and DCs (65). TLR4 activation in these cells induces the release of inflammatory cytokines and chemokines that promote the further recruitment of innate immune cells to the intestinal mucosa (66). Among the receptors involved in the recruitment of immune cells, the chemokine receptor CX3CR1 is responsible for the maintenance of mononuclear cell populations in the lamina propria (67). It has been seen that CX3CR1 deficiency is associated with a reduced number of resident intestinal macrophages in HFD-fed mice. This finding was associated with an elevation in the translocation of bacterial components to the liver (68), demonstrating that innate cell turnover is essential for intestinal homeostasis and lessening the inflammatory damage in NAFLD.

#### Peroxisome proliferator-activated receptor

Due to the absence of pharmacological treatments for NAFLD, diverse receptors involved in controlling metabolic disturbances have been studied, among them the PPAR. Three isoforms of PPAR have been described: PPARα, located mainly in the liver; PPARδ (also known as PPARβ), in the skeletal muscle, adipose tissue, and skin; and PPARγ in adipose tissue (69). Free fatty acids, eicosanoids, and other complex lipids are endogenous ligands for PPAR. Once the ligand is bound, a heterodimeric complex with the nuclear retinoid X receptor (RXR) is formed.

Consequently, the expression of several genes and proteins involved in beta-oxidation, fatty acid absorption, adipogenesis, and adipocyte differentiation is upregulated to control the metabolism of lipids and glucose (70). The role of PPARs in innate and adaptive immunity has been widely described, which suggests that their modulation can be considered a target for NAFLD treatment. Activated PPARs regulate the expression of several inflammatory genes expressed in a wide variety of tissues and immune cells, such as macrophages, DCs, T cells, and B cells (71). Indeed, PPARα signal activation suppresses the inflammatory gene expression mediated by the NF-κB pathway, decreasing inflammatory cytokine secretion by diverse cell types. In addition, PPARα regulates the absorption of fatty acids, beta-oxidation, ketogenesis, and bile acid secretion (72). Based on these mechanisms, PPARα regulates the hepatic metabolism of fats (73) and glucose (74). A protective role of these receptors has been demonstrated for hepatic steatosis in the context of HFD-fed mice. Thus, PPARα KO mice developed more severe steatohepatitis with MCD diet compared to wild-type mice. Even treatment with a potent agonist for PPARα, wy-14643, prevented the accumulation of hepatic triglycerides as well as liver damage in wild-type mice but not the KO group (75). In this regard, PPARα activation prevents the accumulation of triglycerides by increasing the catabolism of fatty acids.

Moreover, regarding the the anti-inflammatory role of PPAR, studies have evidenced the inhibitory effect of the agonist of this receptor on the NF-κB signaling pathway (76). In this regard, Delerive et al. observed in primary human hepatocytes that synthetic PPARα activators, such as wy-14643 and fibrates, upregulate the expression of inhibitor of nuclear factor-kappa B alpha (IκBα) and reduce the binding activity of NF-κB to DNA; however, these effects not were observed in PPARα-null mice (77), supporting the use of PPARα agonists as a potential treatment for inflammatory diseases. Further studies are needed to evaluate the role of PPARα agonists in preventing and reversing the inflammatory damage in NAFLD progression.

Regarding the role of other PPAR isoforms, PPARδ is expressed mainly in the skeletal muscle, which regulates mitochondrial metabolism and beta-oxidation. It is localized in hepatocytes, KCs, and hepatic stellate cells in the liver, preventing inflammation and fibrosis (78). A study evaluating the role of PPARδ using the synthetic agonist GW501516 showed reductions in obesity development in the HFD-fed mouse model caused by improved insulin resistance and the prevention of lipid accumulation in the liver (79).

Furthermore, PPARγ ligands can inhibit the activation of macrophages and the production of inflammatory cytokines such as TNF-α, IL-6, and IL-1β (80). PPARγ is mainly expressed in adipose tissue, regulating adipocyte differentiation, adipogenesis, and lipid metabolism (81). Regarding innate immunity, its activation is involved in macrophage phenotype polarization, from M1 (inflammatory) to M2 (anti-inflammatory) (82). Additionally, PPARγ has also been implicated in colonic inflammation, having been identified as a target of mesalazine, a 5-aminosalicylate (5-ASA) (83). As previously reported, the proinflammatory activation of KCs contributes to the progression of NAFLD. In this regard, Lumeng et al. demonstrated that diet-induced obesity leads to a shift in the phenotype activation of macrophages from adipose tissue from M2 to M1, which contributes to insulin resistance (84) in an HFD-fed mouse model. Indeed, another study showed that HFD induced hepatic steatosis and the local proinflammatory response associated with the M1-predominant profiling of KCs. Macrophages and M1 KC activation are regulated by several transcription factors, among them NF-κB. Transcriptional regulation conduced by increased NF-κB signaling in the liver has been observed in HFD-fed mice, resulting in an M1 proinflammatory predominance. Thus, by PPARγ activation, M1 phenotype activation can be diverted to the M2 phenotype. In this regard, Luo et al. (2017) demonstrated a shift in lipid-induced macrophage polarization through the use of PPARγ agonists from M1 to the M2 phenotype mediated by a direct interaction between PPARγ and NF-κBp65. These findindings allow the conclusion that the PPARγ agonist could improve hepatic steatosis by M1 KC polarization in HFD-fed mice (85). In conclusion, diverse strategies focused on modulating PPARγ activity can be effective approaches to control the innate profiling that conduces the progression of inflammatory liver damage.

### Adaptive immune response

Unlike innate immunity, the adaptive response induces highly specific responses against harmful antigens. The adaptative immune response starts with the antigen presentation process. The professional antigen-presenting cells (APCs), such as DCs, macrophages, and B cells, induce the activation and differentiation of mucosal T cells in the GALT (86). Consequently, the effector and memory CD4^+^ helper T cells and CD8^+^ cytotoxic T cells are generated (87). CD4^+^ T cells differentiate into Th1, Th2, Th17, Th9, Th22, follicular helper T (Tfh), and peripheral (p) Treg cells, while CD8^+^ T cells differentiate into Tc1 and Tc2, Tc17, Tc9, and CD8^+^ reg T cells as major subtypes in response to the cytokine profile that are received during priming (88, 89).

#### Dendritic cells

Increased activation of the adaptive immune response has been shown in NAFLD in both the intestinal and liver compartments. In this regard, intestinal DCs have emerged as essential mediators of immune responses in non-infectious chronic fibro-inflammatory conditions, such as pulmonary fibrosis, inflammatory bowel disease, and acute pancreatitis (90). Indeed, more DCs have been observed in the small intestine in HFD-induced NAFLD mice compared to normal diet-fed mice (91). Additionally, an MCD diet-induced NASH model in rats characterized by induced liver inflammation showed an increased number of macrophages and DCs in the ileal tissue (92). Specific DC subsets that are altered in NAFLD remain to be identified; therefore, further research is needed to distinguish their phenotype, location, and functional properties to identify specific new therapeutic targets. Altogether, this evidence suggests an increased number of APCs in NAFLD, which may correlate to increased lymphocyte activation.

#### T helper lymphocyte subsets

Th1 cells primarily produce IFN-γ and TNF-α, which activate macrophages and conduce cytotoxic CD8^+^ T cell responses, respectively, promoting the elimination of intracellular pathogens such as viruses and bacteria (93). In contrast, Th2 cells produce cytokines such as IL-4, IL-5, IL-9, IL-10, and IL-13, which promote humoral immune responses. A predominance of Th2 responses is observed in pathogenic processes such as allergy (94) and gastrointestinal helminth infections (95). Th17 cells produce IL-17 and IL-22, crucial for host protection against several extracellular pathogens (96). Additionally, Th17 cells secrete IL-22 and granulocyte-macrophage colony-stimulating factor (GM-CSF) to induce neutrophil recruitment (97). Despite Th17 cells playing a relevant physiological role in maintaining populations of commensal bacteria at the gut barrier, they are involved in the progression of many autoimmune diseases and inflammatory disorders (93), among them NASH (98). Th22 cells produce IL-22, IL-13, IL-26, TNF-α, and granzyme B and regulate different antimicrobial proteins produced by intestinal epithelial cells, such as β-defensin 2 (99, 100). It has been shown that Th22 cells may be involved in allergies, autoimmune diseases, intestinal diseases, and tumors (100). Additionally, Th9 cells predominantly secrete the pro-inflammatory cytokine IL-9, which provides immunity against helminths and antitumor immunity (101). pTreg lymphocytes are generated in response to antigen exposure by APCs at the site of inflammation, and their primary function is to promote mucosal tolerance (102). The numerous functions performed by each T cell population highlights its critical role in the intestine based on its specific abilities in controlling intestinal homeostasis, which requires a delicate balance between effector and regulatory responses (103). Furthermore, B cells differentiate into plasma cells that produce immunoglobulins (Igs) in response to direct antigen recognition by surface Igs. The principal Ig secreted by plasma cells is the IgA class, which contributes to intestinal mucosal immunity and barrier function (104).

#### T cell effector response

In addition to the altered number of APCs, an abnormal T cell effector response has been described in animal models and NAFLD patients. In animal models, an HFD has been demonstrated to induce a change in the percentage of intestinal immune T cell populations in diverse murine models. A study by Su et al. showed an increased CD4^+^/CD8^+^ ratio in the PP of rats fed an HFD for 12 weeks (105). Concerning CD4^+^ profile development in NAFLD, an increase in the level of IFNγ-producing Th1 cells has been reported in an HFD-fed mouse model compared to mice fed a normal diet (106). Similarly, Su et al. observed an increase in the Th1 cell proportion of CD4^+^ T cells and a reduction in the Th2 cell proportion of CD4^+^ T cells in the MLNs in the same HFD-fed mouse model (107). Additionally, the number of CD4^+^ and CD8^+^ T cells in the duodenal lamina propria was observed to be lower in NAFLD patients than in healthy subjects, indicating that intestinal immune function is impaired in NAFLD (108). Furthermore, the number of CD4^+^ and CD8^+^ T cells in the duodenal lamina propria was found to be lower in in NAFLD patients than in healthy subjects, indicating that intestinal immune function is impaired in NAFLD (108). In parallel, an increased Th1/Th2 ratio was consistently observed in liver samples, suggesting a relationship between the immune response in the intestine and the liver in NAFLD (107).

The pathogenic role of the Th17 profile response in NAFLD is controversial. An obesity-driven activation of the IL-17 axis is associated with the development and progression of NAFLD (109). Along with it, an increased proportion of Th17 cells in the full CD4^+^ T cell population has been reported in the MLNs in mice fed an HFD for 12 weeks (107). Conversely, a reduced proportion of Th17 cells within CD4^+^ T cells was detected in the small intestinal lamina propria of mice fed an HFD for 10 weeks compared to those fed a normal diet (110). Similarly, in the MLNs, a lower Th17 cell proportion of CD4^+^ T cells was found in the MCD diet-induced NASH mouse model compared to mice fed a normal diet (111). To these controverted findings, we can add that HFD-fed mice present increased IL-17-producing γδ T cells in the small and large intestinal lamina propria (106). Considering that γδ T cells participate in maintaining the integrity of the intestinal barrier by an interaction with enterocytes and other immune cells (112), the inflammatory role of the Th17 profile remains to be elucidated in this pathology. It is noteworthy that a similar dual role of γδ T cells in inflammation has also been reported in some murine models of colitis (113).

#### T regulatory cells

Intestinal Treg cells regulate mucosal immune responses through diverse mechanisms, including cell-to-cell contact suppression and the secretion of soluble suppressive factors, maintaining immune tolerance to dietary and microbiota components (114, 115). In HFD-fed mice, decreased levels of FOXP3^+^ Treg cells in the intestinal lamina propria (106) and a reduced proportion of CD4^+^/FOXP3^+^ cells in the MLNs compared to mice fed a normal diet (107) have been reported. In contrast, the MCD diet-induced NASH mouse model presented an increased FOXP3^+^ Treg cell proportion of CD4^+^ T cells in the MLNs compared to mice fed a normal diet (111). A therapeutic strategy based on oral anti-CD3 mAbs to elicit Treg induction has been explored in NASH treatment (116). This approach is based on the binding properties of the mAbs to the CD3/T cell receptor (TCR complex) of lamina propria T cells that trigger the upregulation of membrane-bound TGF-β and the conversion to the Th3 reg phenotype. Through the release of TGF-β and IL-10, Th3 cells contribute to the tolerogenic intestinal microenvironment (117).

#### Humoral response

Regarding the role of the gut humoral response, an altered density of IgA^+^ cells and full IgA content in the intestine have been observed in NAFLD. In particular, Su et al. observed increased levels of intestinal IgA in the small intestinal fractions of the 12-week scheme HFD rat model, which were associated with an impairment of gut barrier function demonstrated by a high serum level of endotoxin and D-xylose (105). In contrast, Matsumoto et al. demonstrated that MCD diet-fed mice had lower IgA^+^ cell numbers in the ileal and colonic tissues and decreased IgA content in the feces in comparison to a normal diet. Interestingly, these alterations were prevented by adding fructooligosaccharides to the diet, suggesting a feasible prebiotic role that modifies the gastrointestinal microbiota (118). In line with these results, another study reported a decrease in IgA content in the small intestine mucus in HFD-induced NAFLD rats (119). Considering the role of IgA as a soluble factor that controls the load and composition of the microbiota in the lumen, these results suggest that IgA deficiency contributes to the pathogenesis of liver diseases associated with an altered gut microbiota composition (120).

## Gut lymphocyte migration in NAFLD

Intestinal lymphocyte recruitment from the bloodstream depends on sequential events of lymphocyte-endothelial cell adhesion molecule (CAM) interactions (121). Lymphocyte location in various intestinal compartments, such as the lamina propria and epithelia, is determined by specific homing pathways that are conducted by chemokines released by inflamed tissue. Cytokines and co-stimulatory molecules from mature DCs mediate the specific expression of CAMs in lymphocytes during the antigen presentation process. DCs loaded with specific antigens migrate to the secondary lymphoid tissue located proximal to the site of antigen entry, such as Peyer’s patches, MLNs, and ILFs. In the steady state, the intestinal T cells can be activated by retinoic acid-primed intestinal mucosa DCs (CD103^+^) to maintain a permanent lymphocyte pool population, principally in the small intestine. This activation induces the upregulation of α4β7 integrin and CCR9 receptors that are crucial for the migration of the lymphocytes toward the small intestine. Further, CCR9 upregulates α4β7 integrin expression *via* interaction with C-C motif chemokine ligand 25 (CCL25), which is selectively and constitutively expressed by intestinal epithelial cells. α4β7 integrin can bind the mucosal addressin cell adhesion molecule 1 (MAdCAM-1), which is strongly expressed on Peyer’s patches’ high endothelial venules, allowing the entry of lymphocytes from the bloodstream into the lamina propria (122). Despite the scarcity of information about the mechanism that controls the homing to diverse areas of the intestine, the anatomical distribution of lymphocytes is known to depend on the differential expression of CCR9 and retinoic acid availability. In contrast to the small intestine, CD8+ T cell homing to the large intestine involves the chemokine receptors CXCR3 and GPR15 α4β7 but not CCR9 (98, 123). It is worth noting that CXCR3, CXCR6, and CCR5 induce T cell trafficking to the liver to maintain lymphocyte permanency as a liver-resident cell phenotype (109).

**Figure 3:**
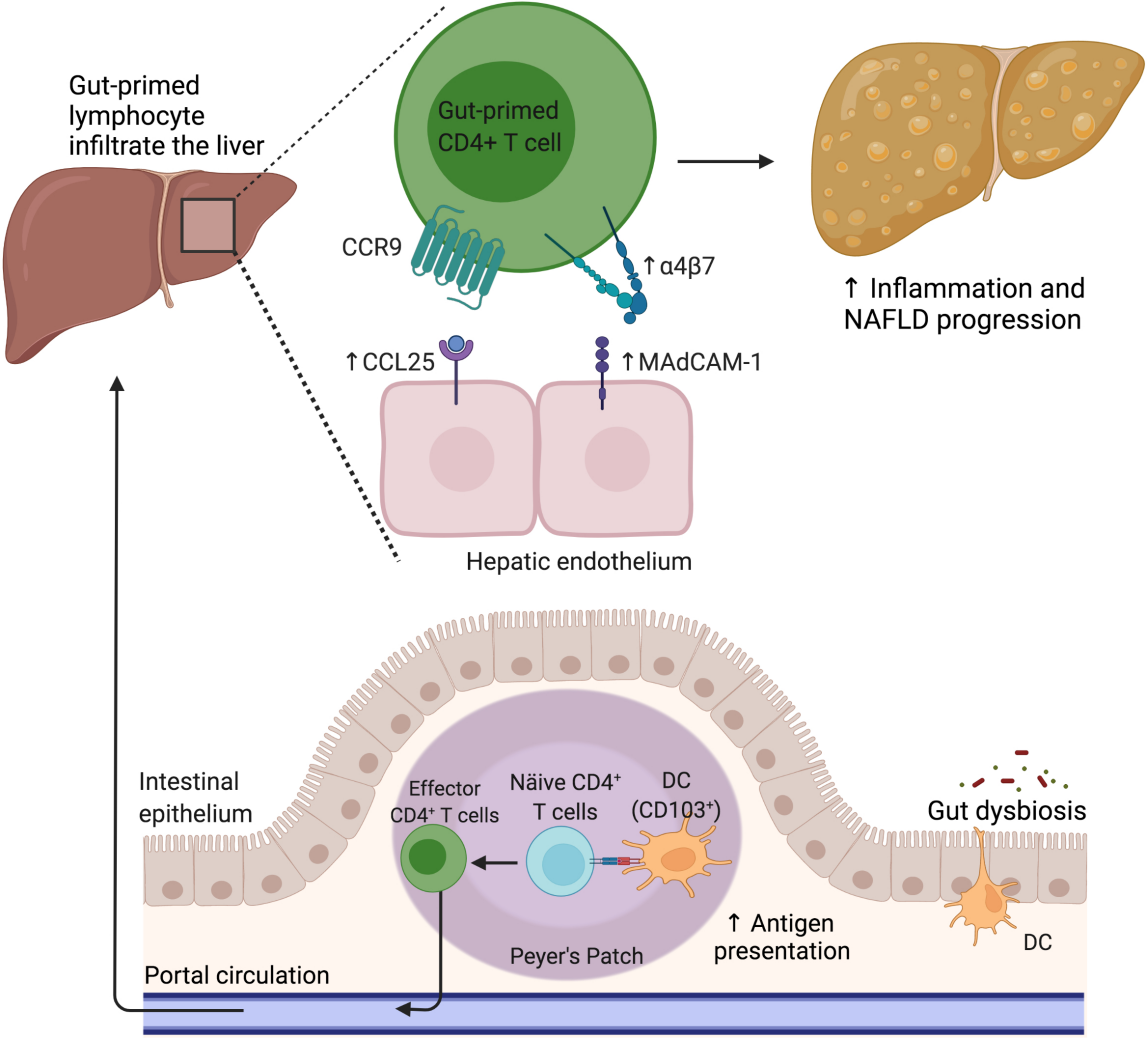
The gut lymphocyte is homing in the NAFLD liver. Under physiological conditions, dendritic cells imprint gut-homing specificity on T cells in the Peyer’s patches or mesenteric lymph nodes by inducing the upregulation of α4β7 integrin and CCR9. Nevertheless, during NAFLD, the hepatic endothelium aberrantly expresses CCL25 and MAdCAM-1, allowing the pathologic recruitment of gut-primed lymphocytes into the liver. CCL25, C-C motif chemokine ligand 25; CCR9, C-C motif chemokine receptor 9; DC, Dendritic cell; MAdCAM-1, Mucosal addressin cell adhesion molecule 1; NAFLD, Nonalcoholic fatty liver disease.

The gut-primed lymphocytes can migrate to extra-intestinal tissues, such as the liver, lung, and skin. This process is highlighted in certain pathological conditions, such as inflammatory bowel disease (IBD) and NAFLD. In this regard, a “gut-lymphocyte homing” hypothesis has been proposed to explain the pathophysiology of NAFLD (**Figure 3**). This concept was initially proposed by Grant et al., who observed a strong association between primary sclerosing cholangitis (PSC) and IBD (124). In this regard, intestinal mucosal T cells were observed in the pool of liver-infiltrating lymphocytes of PSC patients, which were recruited by the aberrant expression of gut-specific CCL25 on the hepatic endothelium. These findings were in agreement with abnormal MAdCAM-1 expression in the hepatic endothelium of IBD patients, especially those with PSC comorbidity (125).

In addition, recently, the MLN has been considered a potential source of liver lymphocytes in NAFLD (107, 126, 127), supporting the hypothesis of gut-lymphocyte homing in this pathology. In this regard, an increased number of CCR9^+^ cells in the liver and an elevated serum CCL25 level were observed in NASH patients in comparison to healthy controls, as well as an increased number of CCR9^+^ macrophages and CCR9^+^ hepatic stellate cells (HSCs) in a high-fat/high-cholesterol (HFHC)-diet mouse model. These findings were associated with liver disease severity and prevented by CCR9 antagonist treatment, suggesting that approaches orientated at blocking the CCR9/CCL25 axis can effectively prevent liver fibrosis progression (128). Furthermore, an increased proportion of Th1 and Th17 in MNL CD4^+^ T cells was observed in HFD-fed NAFLD mice, with increased *in vitro* chemotaxis of MNL CD4^+^ T cells to the liver extract from HFD-fed NAFLD mice (107).

Similarly, a high level of migration to the liver of adoptive-transferred, gut-derived lymphocytes from HFD-induced NAFLD donor mice to NAFLD recipient mice was observed compared to that in control recipient mice. This migration bias was associated with exacerbated liver damage. Upregulated CCL5 expression was observed in the liver of NAFLD recipient mice, along with CCL5 receptor CCR3 in transferred MLN immune cells. The MLN liver migration was inhibited by using a CCL5-blocking antibody *in vitro* (129). Additionally, the increased microbiota-driven intestinal and hepatic expression of MAdCAM-1 contributes to α4β7^+^ CD4^+^ T cell recruitment to the intestine and liver in murine steatohepatitis models (130, 131).

Interestingly, MAdCAM-1 and β7 have been reported to have opposing roles in liver-damaging progression, wherein the upregulation of MAdCAM-1 has been described as being pathogenic in contrast to the expression of β7, which appears to be protective in hepatic inflammation and the liver oxidative response (130). These shreds of evidence suggest that the increased propensity of gut lymphocytes to migrate to the liver in NAFLD can reinforce the inflammatory environment of liver injury. Thus, diverse strategies targeting the molecular mechanisms involved in the migration of activated immune cells in the intestinal environment to the liver, like MAdCAM-1, promise to reduce liver damage progression in this pathology.

## The liver immune response

The contribution of the liver immune response to NAFLD progression has been widely described. In this regard, high activation of resident immune cells, hepatocytes, and sinusoidal endothelial cells plays a significant role in NAFLD pathogenesis by contributing to the chronic inflammatory status. Both innate and adaptive immune responses are involved in the onset of liver damage, reinforcing the recruitment of immune cells, such as monocytes, macrophages, neutrophils, innate lymphoid cells, and CD8^+^ and CD4^+^ T cells, to the site of inflammation (132, 133).

### Innate immunity

The liver tissue is composed of diverse types of cells that are involved in liver immune responses. Hepatocytes constitute 60% of the liver mass and have a metabolic function and depurative role regarding waste products and substances harmful to the organism. In response to diverse stimuli, hepatocytes contribute to innate immune responses through their ability to produce and secrete various inflammatory proteins. Several innate receptors are expressed in hepatocytes. TLRs have been described to have significant roles in NAFLD pathogenesis (134). Elevated circulating LPS levels are observed in diverse NAFLD mouse models, associated with increased intestinal permeability and altered microbiota.

As resident macrophages in the liver, KCs are located in the hepatic sinusoids close to parenchymal and nonparenchymal cells. KCs act as sentinels in the sinusoidal barrier and prevent the spread of filtered products from the intestinal wall to the systemic circulation. KCs exert tolerogenic responses to gut-derived substances, commensal antigens, and death cell products under healthy conditions. Indeed, these cells are permanently exposed to low LPS levels from the gut microbiota that trigger the activation of inflammatory control mechanisms mediated by the release of IL-10 (135). However, under pathogenic conditions, KCs can switch to an inflammatory phenotype, acting as APCs and releasing pro-inflammatory mediators (cytokines, prostanoids, nitric oxide, and oxidizing agents) that contribute to liver inflammation (136–138). Increased activation of TLR4 signaling has also been observed for KCs during NAFLD development, with TLR4-mutant mice being resistant to this damaging process (139). In this regard, massive TLR4 stimulation in response to increased LPS levels from the portal circulation leads to KCs producing several chemokines, such as CCL2 (10). Likewise, a significantly increased expression of CCL5 was detected in the liver of HFD-fed mice, associated with significant hepatic steatosis (140). These findings agree with the increased CCL5 mRNA expression observed in liver samples from patients with fibrotic NASH compared with subjects with simple steatosis, suggesting that CCL5 expression is a marker of fibrotic liver disease (141). Both CCL2 and CCL5 are involved in the recruitment of lymphocytes to the liver (10). It has been shown that CD4^+^ T and CD8^+^ T cells activated in the MLNs can migrate to the liver, leading to liver injury, while CCL5 blockade prevents the recruitment of T cells. These findings suggest a crucial role for CCL5 in the migration of gut-derived lymphocytes in the NAFLD mouse model (129).

Additionally, augmented infiltration of LY6C2^+^ monocytes into the liver has been described, mediated by the CCR2–CCL2 interaction. This event is considered critical in steatohepatitis development and subsequent fibrosis progression (10, 142). A pro-inflammatory M1 phenotype can be induced in KCs by liver metabolic abnormalities. Under this condition, KCs perpetuate the effects of a high-fat diet by increasing triglyceride accumulation in hepatocytes and decreasing fatty acid oxidation and insulin responsiveness, which is attenuated by the neutralization of TNFα *in vitro*. Considering that the depletion of KCs limits the development of liver inflammation, insulin resistance, and alterations in hepatic lipid metabolism and fibrosis, this evidence suggests that KCs are a crucial intermediary in the cross-talk of immune-metabolic liver functions (143).

Natural killer (43) cells and natural killer T (NKT) cells are relevant components of the innate immune response in NAFLD. Both cells have a significant role in the progression of liver damage due to their cytotoxic impact and the promotion of pro-inflammatory responses, demonstrated in viral hepatitis and chronic liver inflammation (144). Liver-resident NKs possess immunophenotypic and functional characteristics that differ from peripheral NKs, sharing functional properties with the innate lymphoid cells of mucosal tissues (145). NKT cells reside in the sinusoids, playing an immune surveillance role, acting as sentinels, and eliminating pre-malignant senescent hepatocytes. Two subsets of NKTs have been described: a proinflammatory type I phenotype, principally activated by lipids, and type II, abundant in the livers of both mice and humans compared to the type I subtype. NK T cell types I and II have opposing immune functions, with a protective role described for type II cells in liver inflammation (146). Interestingly, HFD-fed NKT cell-deficient mice, lacking the NKT type I and II phenotypes, are prone to developing diet-induced obesity and metabolic perturbations due to increased inflammatory responses and steatosis in the liver (147). Unlike these effects, the systemic depletion of NK cells in an HFD mouse model of obesity decreased macrophage infiltration into the adipose tissue, reducing systemic inflammation and insulin resistance (148). This evidence suggests that NKT cells may be a therapeutic target in modulating metabolic disorders in the liver, among them NAFLD. Unlike NKT cells, NK cells’ role in metabolic disturbances, liver inflammation, and damaging progression remains controversial.

Lipotoxic lipid species induce hepatocyte damage and the release of neutrophil-recruiting chemokines, such as CXCL1 and IL-8 (149). The increased liver infiltration of neutrophils in NAFLD initiates and enhances inflammation, reinforcing the recruitment of macrophages and the interaction with APCs. Changes in neutrophil granular content and composition have been associated with diverse inflammatory processes in several NASH models (149). Myeloperoxidase (MPO), a pro-oxidant enzyme released by neutrophils, enhances macrophage cytotoxicity and promotes inflammation and fibrosis in HFD-fed mice. In contrast, MPO-deficient mice attenuate NASH development (150). Additionally, neutrophils promote insulin resistance and inflammation by releasing neutrophil elastase levels in HFD-fed mice (151). This evidence shows that the increased infiltration and activity of neutrophils contribute to the inflammatory damage in and progression of NAFLD. As for human models, increased neutrophils and elevated MPO plasma levels have been observed in patients with NASH compared with those with fatty liver. Thus, MPO activity in the liver is associated with increased inflammation in these patients (152).

Mast cells (MCs) have been highlighted as important regulators of pathogenic processes in liver disease progression (153). In NASH patients, the number of hepatic MCs positively correlates with the stage of fibrosis (154). This evidence suggests that the modulation of MCs may be another attractive therapeutic target for treating NASH.

### Adaptive immunity

Regarding adaptive immune responses, it has been demonstrated that DC activation and its immune phenotype commitment are essential for the perpetuation of liver damage (127). Among the adaptive immune cells, DCs are responsible for initiating and limiting liver inflammation through their properties of presenting antigens to lymphocytes in the neighboring lymphoid organs as well as eliminating apoptotic and necrotic waste. The liver contains several types of DCs that are usually located surrounding the central veins and the portal system. In the normal liver, resident DCs exhibit an immature phenotype imprinted by a tolerogenic IL-10-enriched microenvironment (155). Under inflammatory conditions, DCs are recruited from the hepatic sinusoids to periportal areas. LPS and peptidoglycan induce the upregulation of costimulatory molecules, such as CD40, CD80, and CD86, and the release of inflammatory cytokines by hepatic DCs through TLR4/MD2 complex activation (156). The role of hepatic DCs in NAFLD pathogenesis is controversial due to liver population heterogeneity and differences in the mouse models of disease used in various studies (157). Although murine CD103^+^ DC subtype (classical type-1 DCs and cDC1s) influences the pro-and anti-inflammatory balance and protects the liver from metabolic and inflammatory damage (158), increased activation and abundance of these cell types have been observed in NAFLD patients, promoting inflammatory T cell reprogramming in NASH (159).

Regarding the role of liver B and T lymphocytes in NAFLD, increased infiltration of B2 cells and CD4^+^ and CD8^+^ T cells as well as elevated circulating antibodies have been observed in NASH (133). In terms of T cell subsets, an increase in Th1 and Th17 cells and a reduction in Treg levels in the liver have been observed in NAFLD patients compared to healthy controls, principally in patients with steatohepatitis compared with those with simple steatosis (133). Also, Th1, Th17, and CD8^+^ lymphocytes contribute to hepatic macrophage activation and NKT cell recruitment in NASH murine models (133). Additionally, increased γδT cell recruitment to the liver has been associated with the progression of steatohepatitis, mainly due to the IL-17-secreting subset (160). Considering that CD4, CD8, and γδ T cells can recognize microbial peptides and lipid antigens, this suggests that the overall stimulation of the intrahepatic T cell subsets can be directly influenced by the homeostasis of the gut microbiota (161).

Moreover, recent evidence supports that gut microbial factors drive the pathogenic function of B cells during NASH development. Fecal microbiota transplantation from humans with NAFLD into recipient mice induces the increased accumulation and activation of intrahepatic B cells, predominantly pro-inflammatory IgM^+^ IgD^+^ B2 cells (162). These findings suggest that the adaptive immune response is highly activated in NAFLD, associated with a decreased tolerogenic response and disease progression.

## Treatments for NAFLD: Targeting the immune response

The current treatment for NAFLD focuses on improving patients’ lifestyles by promoting a healthy diet, physical activity, and weight loss. Despite compelling evidence of the effectiveness of these recommendations in reducing liver damage and even reversing liver fibrosis (163), low treatment adherence leads groups of patients to search for therapeutics directed at complementing current clinical protocols.

The first two drugs evaluated for NAFLD treatment were vitamin E and pioglitazone, an antidiabetic agent. Although there is diverse evidence of their beneficial effect on liver function and NASH resolution (164–166), the use of these drugs is limited due to the high risk of side effects under prolonged administration. Recently, two not yet fully approved hypoglycemic drugs, glucagon-like peptide-1 (GLP-1) agonists and sodium-glucose cotransporter-2 (SGLT-2), showed effectiveness in reducing liver inflammation and fibrosis, mainly in a subgroup of diabetic patients in phase II and III trials (167, 168). Moreover, different therapeutic approaches for liver diseases are focused on modulating the gut microbiota, including prebiotics and probiotics (169). The results of their evaluations support the benefit of this complementary strategy in NAFLD pharmacological therapy (170, 171).

There are several potential strategies to normalize altered hepatic metabolism in NAFLD. These include therapies aimed at reducing hepatic steatosis by modulating the lipid metabolism. BAs have been described as promising alternatives in NAFLD treatment. Since BAs regulate carbohydrate and lipid metabolism in the liver, approaches directed at increasing their function have resulted in an interesting strategy to reduce the liver metabolic overload and liver damage in NAFLD. By interacting with their nuclear receptor FXR, BAs can activate the gene expression of diverse components involved in the entero-hepatic metabolic pathways (172). Obeticholic acid (OCA), an analog of chenodeoxycholic acid, is a semi-synthetic BA with high affinity and selectivity for FXR that has been trialed in NASH. Along with FXR activation in the ileum, which induces the secretion of fibroblast growth factor 19 (FGF-19) and its subsequent transport to the portal system, OCA decreases the production of BAs, stimulates beta-oxidation, and decreases lipogenesis and gluconeogenesis in the liver (173). Randomized studies in patients with NASH have shown that OCA significantly improves steatosis, inflammation, ballooning, and liver fibrosis compared to placebo, with a low rate of side effects such as pruritus and an increase in LDL being the most frequent (174). These studies are consistent with previous multicenter, randomized, placebo-controlled trials in NASH patients (FLINT and REGENERATE) in which improvements in liver histology, including a decrease in the fibrosis stage, were achieved by the use of OCA (53, 175). The therapies described above are among those most frequently addressed in NASH. However, the modulation of the immune response has been little discussed in these studies.

The modulation of the immune response has been a secondary aim in NAFLD therapy since the main approaches are focused on reducing liver fat loading to lessen inflammation. However, few works focus on controlling the immune response at the intestinal level because most studies have been directed at the immune response in the liver. Thus, possible targets in the intestinal response would be interesting to study. To classify the immune targets explored in NAFLD, we have divided the strategies into treatments focused on controlling inflammation, including modulating agents of the innate immune response and receptors associated with inflammatory pathways, and those that control the adaptive immune response (**Table 1**).

**Table 1:** Clinical trials of pharmacological approaches targeting the immune response in NAFLD.

Target	Drug	Mechanism of action	Phase	NCT number
**Inflammation control**				
PRR antagonist	JKB-121	Antagonizes the TLR4 receptor Prevents LPS-induced inflammatory liver injury Inhibits hepatic stellate cell proliferation and collagen expression	Phase 2	NCT02442687
PPARγ agonist	Pioglitazona	Improves steatosis, inflammation, and fibrosis Increases insulin sensitivity Regulates lipid production and metabolism	Phase 3	NCT00063622
PPARα/δ/γ agonist	Lanifibranor	Modulates the inflammatory, metabolic, and fibrogenic pathways Improves fibrosis, inflammation, and steatosis Decreases liver enzymes	Phase 2b	NCT03008070
PPARα/δ agonist	Elafibranor	Reduces liver enzymes, steatosis, and markers of systemic inflammation	Phase 2b	NCT01694849
ASK1 inhibitor	Selonsertib	Reduces liver fibrosis Improves lobular inflammation	Phase 2	NCT02466516
Anti-LPS	IMM-124E	Polyclonal antibody mixture specific to LPS in the gastrointestinal tract Did not produce any evidence of clinical benefit and was not able to reduce the fat content of the liver in NASH patients	Phase 2	NCT02316717
**Adaptive response control**				
Chemokine receptor inhibitor	Cenicriviroc	Anti-fibrosis Reduces inflammatory cell recruitment Inhibition of CCR2–CCR5 reduces short-term fibrosis progression	Phase 2b	NCT02217475
Cytokine inhibitors	Pentoxyfylline	Inhibition of a number of pro-inflammatory cytokines including TNF-α Improves steatosis and lobular inflammation	Phase 2	NCT00590161
Antigen recognition/Treg induction	Oral anti-CD3 mAb	Binds T lymphocytes in the gut and modulates the CD3/T cell receptor, eliminating T cell proliferation and the release of proinflammatory cytokines. Treg induction in the intestinal lamina propria	Phase 3	NCT03291249

ASK1, Apoptosis signal-regulating kinase 1; CD, Cluster of differentiation; LPS, Lipopolysaccharide; mAb, Monoclonal antibody; NAFLD, Nonalcoholic fatty liver disease; NASH, Nonalcoholic steatohepatitis; NCT, National Clinical Trial; PPAR, Peroxisome proliferator-activated receptor; PRR, Pathogen recognition receptor; TLR, Toll-like receptor; Treg, Regulatory T cell.

### Innate immune targeting approaches: The control of inflammation

The liver is exposed permanently to gut-derived endotoxins, such as LPS, that enter the enterohepatic circuit after passing across the intestinal epithelial barrier. LPS in the liver activates KCs by TLR4 signaling, provoking pro-inflammatory gene expression and the subsequent release of mediators that induce hepatic injury and fibrosis. Thus, strategies that block the TLR4 pathway appear promising to prevent the progression of liver inflammatory damage. A preclinical study in mice showed that KC depletion by the intravenous injection of clodronate liposomes reduced histological evidence of steatohepatitis and prevented the increase of TLR4 expression in the liver, which demonstrates that the link between KCs and TLR4 signaling plays a central role in the pathogenesis of steatohepatitis (176). Other studies also assessed this approach and focused on evaluating the impact of TLR4 signaling antagonists on liver damage. JKB-121, a non-selective opioid TLR4 antagonist, has been shown to prevent LPS-induced inflammatory liver injury in an MCD diet-fed rat model of NAFLD (177). Additionally, *in vitro* experiments have shown that JKB-121 could reduce the release of LPS-induced inflammatory cytokines and inhibit hepatic stellate cell activation (178). However, a randomized, double-blind, placebo-controlled phase II trial of JKB-121 showed unsatisfactory results, wherein JKB-121 did not improve the liver fat content and liver fibrosis biomarkers in patients with NASH compared to placebo (NCT02442687) (179). Given the multiple relevant biological pathways of TLR4 in the pathogenesis of NAFLD, further investigation of TLR4 inhibition is necessary (NCT02442687). In line with this evidence, a phase II study in NASH patients using a polyclonal antibody mixture specific to LPS and other pathogenic bacterial components (IMM-124E) did not produce any evidence of clinical benefit. The intervention could not reduce the liver fat content but decreased serum LPS levels and AST and ALT biomarkers associated with liver function (180).

Another therapeutic approach studied for NASH treatment is the inhibition of the activation of apoptosis signal-regulating kinase 1 (ASK1). Under pathological conditions, an increase in oxidative stress in hepatocytes induces ASK1 autocleavage, leading to increased p38/JNK pathway activation that worsens hepatic inflammation, apoptosis, and fibrosis (181). Selonsertib, a selective inhibitor of ASK1, was evaluated in a phase II clinical trial for NASH (NCT02466516) and improved liver fibrosis and decreased fibrosis progression rates over a 24-week treatment period, indicating its potential as an anti-fibrotic therapy (181). Specifically, 18 mg of selonsertib was shown to lead to improvements in 43% of patients in at least one stage of fibrosis, compared to 30% for selonsertib 6 mg. However, other phase III studies using selonsertib in NAFLD did not achieve the expected results at week 48 of treatment and were terminated (NCT03053050 and NCT03053063).

As discussed above, the role of PPARs in regulating the liver immune response has been well described. Multiple studies have been conducted to modulate PPAR nuclear activity by using receptor agonists. In a phase IIb study, elafibranor, a PPARα/δ dual agonist, resolved NASH after 52 weeks of treatment by reducing liver enzymes, steatosis, and systemic levels of inflammatory markers (NCT01694849) (182). Another phase III study (NCT02704403) using elafibranor showed reduced liver fibrosis stages in the subgroup of patients that reached NASH resolution compared to the group without NASH resolution; however, this study was suspended because it did not meet the primary endpoint. The drug did not worsen the fibrosis, but only 24.5% of patients who received elafibranor 120 mg achieved fibrosis improvement of at least one stage compared to 22.4% in the placebo group. In this regard, in a phase IIb trial, the pan-PPAR agonist lanifibranol demonstrated effectiveness in modulating inflammatory, metabolic, and fibrogenic pathways in NAFLD pathogenesis in patients with non-cirrhotic NASH who were treated for 24 weeks (183).

Since obesity is associated with the induction of pro-inflammatory profiling in gut-immune populations, anti-inflammatory therapies targeting the gut, such as mesalazine (5-aminosalicylate, 5-ASA), have been investigated in HFD-fed mouse models. Treatment with mesalazine reduced liver steatosis compared to the non-treated group (106). This finding suggests that controlling the inflammatory response in the intestine contributes to the lessening of the liver damage induced *via* the accumulation of fatty acids in the liver.

### Adaptive immune response-based approaches

The adaptive immune response plays an essential role in liver damage due to the recruitment and migration of cells to the liver, leading to the generation of proinflammatory immune profiles responsible for the increased oxidative and inflammatory damage in NASH. Therapeutic strategies focused on modulating the activation of the adaptive immune response in the gut have been explored (116). An oral anti-CD3 monoclonal antibody treatment induced intestinal regulatory T cells that suppress the chronic inflammatory state associated with NASH. The anti-CD3 monoclonal antibody foralumab is currently in phase II of development (NCT03291249).

In NASH, several studies have focused on chemokine blockage to prevent the elevation of immune cell recruitment in the liver. Cenicriviroc is a dual antagonist that inhibits the CCR2/CCR5b chemokine receptors. This experimental drug can potently block the infiltration of pro-inflammatory monocytes and macrophages *via* the antagonism of CCR2. It also has antifibrotic activity in the liver due to the modulation of immune cells and hepatic stellate cells *via* CCR5 inhibition. A phase IIb multinational, randomized, double-blinded, placebo-controlled study has shown that patients with NASH treated with oral cenicriviroc had reduced circulating biomarkers of systemic inflammation, such as high-sensitivity C-reactive protein, IL-6, fibrinogen, and IL-1ß, as well as reduced monocyte activation, compared with the placebo group. These results suggest that cenicriviroc exhibited anti-inflammatory and anti-fibrotic effects at Year 1; specifically, twice as many subjects on cenicriviroc achieved improvement in fibrosis of ≥1 stage and no worsening of steatohepatitis compared to those on placebo (184). Despite the promising results obtained in the phase II trial, the phase III clinical study was interrupted early due to lack of efficacy (NCT03028740).

## Discussion

The pathogenesis of NAFLD is complex and directly related to metabolic syndrome. Environmental factors including diet, lifestyle, obesity, insulin resistance, and psychosocial stress determine its development based on the individual characteristics of susceptibility, genetics, gut microbiota, and immune response of the patients. Metabolic syndrome establishment has several consequences in the tissues, such as increasing serum free fatty acid and cholesterol levels, leading to adipocyte dysfunction. This syndrome has hepatic implications due to the over-accumulation of free fatty acids, leading to lipotoxicity, the release of pro-inflammatory cytokines, and steatosis.

Gut-derived metabolites, whether dietary or microbial, are transported *via* the portal vein to the liver, continuously exposing this organ to potential antigens. Conversely, liver-derived factors, such as BAs, are transported to the gut, influencing gut microbiota composition and function. Liver diseases, including NAFLD, are associated with compositional and functional alterations of the gut microbiota, known as dysbiosis. Dysbiosis impacts the host’s immune and metabolic systems and intestinal barrier integrity. Metabolic mechanisms include effects on glucose and lipid metabolism, mainly mediated by changes in BA composition and alterations in the production of SCFAs. Immune mechanisms include delicate crosstalk between the gut microbiota, intestinal epithelial cells, and gut mucosal system. The disruption of this crosstalk leads to alterations in the modulation of inflammasome signaling through microbial metabolites, activation of TLRs and NLRs, and the shifting of the balance between regulatory and pro-inflammatory T cells. Concerning the intestinal barrier, dysbiosis disrupts its integrity, causing a leaky gut and the increased translocation of microbial components to the MNLs and the GALT. Additionally, when the intestinal barrier is compromised, the liver becomes overloaded with metabolites from the gut, leading to a loss of liver tolerance. Antigens such as LPS derived from the microbiota induce inflammation by binding to the TLRs of KCs. Signaling *via* TLRs leads to pro-inflammatory changes in the liver, and failure to regulate gut microbiota results in further disease progression.

The elevation in gut inflammation and the adaptive immune cell priming at the lymphoid tissue causes aberrant homing of gut lymphocytes to the liver, reinforcing the inflammatory damage of hepatocytes. Gut lymphocyte homing was initially described in NAFLD as involving the aberrant expression of homing receptors in the liver and increased hepatic oxidative stress and inflammation. Nevertheless, the mechanisms of gut immune cell homing to the liver, the immune profiles, and the specific composition of the subsets of these populations have not been fully described. In addition, the characterization of this mechanism should consider the different stages of NAFLD and their association with changes in the gut microbiota composition. It is worth noting that although these mechanisms reinforce and promote damage to the liver (from gut to the liver), whether the deterioration of liver functions implies a metabolic alteration that may affect the synthesis of proteins necessary for the structure of the intestinal epithelial TJs and the mechanisms of regulation of the systemic inflammatory response has not been studied. We propose that the study of gut mucosal immunity in the context of NAFLD could provide novel insights into the development and progression of this disease. In this context, the modulation of intestinal mucosal immunity stands out due to its direct relationship with the liver.

Despite several advances in the understanding of the pathophysiology underlying NAFLD, no drugs have been approved by the Food and Drug Administration (FDA) to treat either simple steatosis or NASH. Thus, no specific therapy can be firmly recommended, and any drug treatment would be off-label (185). Clinical trials have focused mainly on the metabolism as a target, while studies that focus on drugs whose primary target is the modulation of the immune response are scarce. Interestingly, gut lymphocyte homing is a process involved in NAFLD pathogenesis, as has been shown in the experimental NASH model where MAdCAM-1 deficiency improved the disease (130); therefore, further research aimed at evaluating the pharmacological modulation of gut lymphocyte homing as a therapeutic strategy is needed.

Based on this revised information, we propose that modulating intestinal inflammatory events at the onset of the disease could be a possible therapeutic target that has remained unexplored thus far. Further research is needed to fill the gaps in knowledge of the immunological mechanisms altered in NAFLD to identify specific new potential therapeutic targets.

## A perspective on future research

To date, the primary treatment for NAFLD is based on metabolic control measures, such as weight loss or exercise. However, these medical indications require high patient adherence, which is not always reached. Current research in this area has allowed a greater understanding of the pathogenesis of the disease, giving rise to new treatment options. As we described, the altered immune response in NAFLD is associated with the dysfunction of the gut-liver axis. Indeed, increased immune cell activation plays a crucial role in the onset of the disease and the course of chronic liver damage. Based on this, determining inflammatory mediators and identifying activated immune cell profiles could significantly contribute to the determination of predictive biomarkers of NAFLD progression toward NASH or cirrhosis. On the other hand, new research focused on describing immune response dynamics in this pathology will allow us to propose new therapeutic targets for the modulation of adaptive immunity. In this regard, it is essential to highlight the role of increased gut-to-liver lymphocyte homing in the pathogenesis of NAFLD. In this regard, therapies based on the blockage of activated lymphocyte migration, such as CCR9 antagonists, could reduce lymphocytic infiltration in the liver and prevent tissue damage that leads to fibrosis progression.

## Author contributions

NO-L, CF, MD and AP-L: drafting the original manuscript. NO-L, CF, MD, LV-P, and CB conceived the idea and the aim of the review. CB, LV-P, AP-L, AE, JR and JP reviewing. CB, AE and LV-P: editing. NO-L, CF, MD, AP, AE, JR, JP, LV-P, and CB: validation. CB and LV-P: supervision. CB: funding acquisition. All authors contributed to the article and approved the submitted version.

## Funding

Financed by FONDECYT 1181699, ANID.

## Conflict of interest

The authors declare that the research was conducted in the absence of any commercial or financial relationships that could be construed as a potential conflict of interest.

## Publisher’s note

All claims expressed in this article are solely those of the authors and do not necessarily represent those of their affiliated organizations, or those of the publisher, the editors and the reviewers. Any product that may be evaluated in this article, or claim that may be made by its manufacturer, is not guaranteed or endorsed by the publisher.
